# Association study of polymorphisms in *FOXO3, AKT1* and *IGF-2R* genes with human longevity in a Han Chinese population

**DOI:** 10.18632/oncotarget.6627

**Published:** 2015-12-16

**Authors:** Ning Li, Huaichao Luo, Xiaoqi Liu, Shi Ma, He Lin, Rong Chen, Fang Hao, Dingding Zhang

**Affiliations:** ^1^ Sichuan Provincial Key Laboratory for Human Disease Gene Study, the Institute of Laboratory Medicine, Sichuan Academy of Medical Sciences and Sichuan Provincial People's Hospital, Chengdu, Sichuan, China; ^2^ Department of Immunology, Zunyi Medical College, Zunyi, Guizhou, China; ^3^ Sichuan Translational Medicine Hospital, Chinese Academy of Sciences, Chengdu, Sichuan, China; ^4^ Department of Health Management, Sichuan Academy of Medical Sciences and Sichuan Provincial People's Hospital, Chengdu, Sichuan, China

**Keywords:** human longevity, FOXO3, AKT1, IGF-2R, single nucleotide polymorphism (SNP), Gerotarget

## Abstract

FOXO3, AKT1 and IGF-2R are critical members of the insulin/IGF-1 signaling pathway. Previous studies showed that polymorphisms (SNPs) in FOXO3, AKT1 and IGF-2R were associated with human longevity in Caucasian population. However, the association of these SNPs in different ethnic groups is often inconsistent. Here, we investigated the association of genetic variants in three genes with human longevity in Han Chinese population. Twelve SNPs from FOXO3, AKT1 and IGF-2R were selected and genotyped in 1202 long-lived individuals (nonagenarians and centenarians) and younger individuals. Rs9486902 of FOXO3 was found to be associated with human longevity in both genders combined in this study (allelic P = 0.002, corrected P = 0.024). The other eleven SNPs were not significantly associated with human longevity in Han Chinese population. The haplotypes TTCTT, CCTTC and CTCCT of FOXO3 as well as GGTCGG and GGTCAG of AKT1 were shown to have a significant difference between case and control (P =0.006, 2.78×10^−5^, 4.68×10^−6^, 0.003,0.005, respectively). The estimated prevalence of diabetes and prediabetes in long-lived individuals was significantly lower than in common adult populations (P = 0.001, 2.3×10^−26^). Therefore, the search for longevity-associated genes provides the identification of new potential targets beneficial for the treatment of diabetes.

## INTRODUCTION

The problem of aging population is known as one of the severest problems in developed and developing countries. In 2014, it is estimated that the number of elders (65 years old and over) was two hundred million in China. This figure will be three hundred million in 2025, and is still rising. The majority of age-related chronic diseases will afflict elders, such as type 2 diabetes, hypertension, coronary heart disease, cancer, etc. Previously, Terry et al. [1] reported that long-lived individuals (nonagenarians and centenarians) will delay or escape from age-related disease. However, the exact molecular basis of this phenomenon is scarcely known. Thus, identification of longevity related gene/loci is not only defining the underlying mechanisms of human longevity, but also to provide insights into the study of pathogenesis of age-related disease in the future.

In the last few years, several studies have shown that many SNPs in genes from the insulin/IGF-1 signaling pathway were significantly associated with human longevity. For instance, *FOXO3*, *AKT1* [2], *FOXO1A* [3], *INS1* [4], *INSR* [5], etc. Willcox et al. [6] showed that the polymorphisms of the *FOXO3* gene (rs2802292, rs2764264, rs1327795) were significantly different between long-lived individuals and controls with *P* value of 0.0002, 0.0006, 0.0001 respectively, suggesting that these variations were more likely to be susceptibility polymorphism of human longevity in a Japanese population. In addition, a considerable number of replicated studies were frequently reported. These studies include rs2802292 in *FOXO3* (Li et al. 2009; Anselmi et al. 2009; Soerensen et al. 2010) [7–9], rs 2764264 in *FOXO3* (Anselmi et al. 2009; Soerensen et al. 2010) [8, 9], rs13217795 in *FOXO3* (Soerensen et al. 2010) [9], rs4946936 in *FOXO3* (Flachsbart et al. 2013; Li et al. 2009) [7, 10], rs7762395 in *FOXO3*(Soerensen et al. 2010; Nygaard et al. 2014) [9, 11], rs1935949 in *FOXO3* (Pawlikowska et al. 2009) [12], rs479744 in *FOXO3* (Li et al. 2009; Soerensen et al. 2010; Flachsbart et al. 2009) [7, 9, 13]. However, it is unclear whether these loci recently identified in Japanese and Europeans exert a similar effect on human longevity in a Han Chinese population in the region of Sichuan. Li et al identified that genetic variants in *FOXO3* are significantly associated with human longevity in the Han Chinese population. Then, Zeng et al also confirmed the joint effects of the genotypes of *FOXO3* and *FOXO1A* genes on human longevity [7, 14]. *FOXO3* and *FOXO1A* are critical downstream molecules of *AKT1* gene [15]. Thus, we hypothesized that similar to *FOXO3*, *AKT1* gene also possibly exerted effects on human longevity. Pawlikowska et al. [12] reported that the polymorphism of the *AKT1* gene (rs3803304) has been significantly associated with human longevity in a meta-analysis across the three cohorts that consist of the cardiovascular Health study, the study of Osteoporotic Fractures and Ashkenazi Jewish Centenarians. In contrast, Nygaard et al. [16] showed that rs3803304 in the *AKT1* gene was found not to be associated with human longevity in Danish and German long-lived individuals. Remarkably, these results present a paradox. In addition, Yousin Suh et al. [17] found that genetic variants in the human *IGF-1R* that result in altered IGF signaling pathway increase the susceptibility of human longevity. Soerensen et al. [18] demonstrated that SNP rs9456497 in the *IGF-2R* was significantly associated with human longevity in a Danish population, composed of 1083 long-lived individuals and 736 middle aged controls. However, this result has not been replicated by other cohorts. Meanwhile, the association between *AKT1* and *IGF-2R* with longevity has not been investigated in the Han Chinese. In the present study, we first investigated whether genetic variants in *AKT1* and *IGF-2R* genes are associated with longevity in a Han Chinese population.

Functional studies showed that *FOXO3* was involved in insulin resistance, several kinds of cancer and Type 2 diabetes [19]. *FOXO3* was also important for the onset of diabetic cardiomyopathy and associated with hemoglobin A1c level and fasting plasma insulin [19, 20]. Although Soerensen et al reported that genetic variants in *FOXO3* were not significantly associated with self-reported diabetes in oldest-old Danes, the *FOXO3* protein has a wide array of downstream targets, which themselves affect a wide range of cellular and physiological processes [21]. Thus, its molecular mechanism still remains uncertain. Considering the fact that gender and ethnic population have effects on the human longevity, gender stratification and the classification of lineages of long-lived individuals were necessary. In the present study, first, we analyzed Y chromosome haplogroups lineage distribution of long-lived individuals from a Han Chinese population in the region of Sichuan. Second, we examined the relationship between the 12 SNPs of *FOXO3*, *AKT1* and *IGF-2R* with human longevity in a mainland Han Chinese population. Third, we compared the prevalence of diabetes and prediabetes of long-lived individuals in our study with a common adult population reported by Xu et al. [22]. We demonstrated that rs9486902 in *FOXO3* gene is associated with human longevity in both genders, and the estimated prevalence of diabetes and prediabetes were significantly lower in long-lived individuals than in common adult population in this Han Chinese population.

## RESULTS

### Population characteristics

In this study, long-lived individuals and the younger control individuals were recruited from the region of Sichuan in the southwest of China. According to Y chromosome haplogroups lineage distribution [23], the region of Sichuan includes Y chromosome haplogroup N* 8.8%, O3-M122 80%. Long-lived individuals underwent a standard set of laboratory tests that included measurements of hemoglobin A1c levels to assess the control situation of blood glucose levels (Tables 8, 9). The younger controls were not diabetes susceptible populations. Thus, hemoglobin A1c level was not tested in the younger control population.

**Table 1:** The characteristics of long-lived individuals and younger individuals

	Case(n = 576)	Control(*n* = 626)
Gender(male/female)	221/355	282/344
Female (%)	61.6	55.0
Mean age(age)^a^	92.8±2.6	34.1±4.1
Age range(age)	90-109	20-38

a The age when the cases and controls were recruited, ±: Standard deviation

### Genotype association analysis

Rs9486902 in the *FOXO3* gene was shown to be significantly associated with human longevity in the present study population with genotype frequencies of long-lived individuals and of younger controls (*P* = 0.008, Table 2), but after bonferroni correction, the significant associations no longer existed (corrected *P* = 0.096, Table 2). To further evaluate the association between *FOXO3*, *AKT1*and *IGF-2R* with longevity, we applied four different genetic models. Rs9486902 showed highly significant association in Addictive 2 and Dominant models (*P* = 0.003, OR= 0.55, 95%CI 0.82-0.38; *P* = 0.002, OR= 0.55, 95%CI 0.81-0.37, Table 4). Rs9400239 showed borderline significant association in Addictive 1 and Recessive models (*P* = 0.04, OR= 0.66, 95%CI 0.98-0.44; *P* = 0.05, OR= 0.68, 95%CI 0.82-1.27, Table 4). For the multiple correction, the *P*-value for significant observation should be 0.05/(12 SNP x 4 models) = 0.001, because 4 statistical models were applied. As a result, none of twelve SNPs showed a significant difference between case and control after multiple testing.

**Table 2:** Genotype analyses of the 12 SNPs on the *FOXO3, AKT1* and *IGF-2R* in long-lived individuals and younger individuals

Gene	SNP	Genotype	Case n (%)	Control n (%)	*P*	Corrected *P*^a^
*FOXO3*	rs13217795	CC/CT/TT	63(11.0)/224(38.9)/286(49.7)	54(8.6)/237(37.9)/323(51.6)	0.39	
*FOXO3*	rs2464264	GG/AG/AA	62(10.8)/231(40.1)/282(49.0)	53(8.4)/244(39.0)/319(51.0)	0.38	
*FOXO3*	rs9400239	TT/CT /CC	67(11.6)/229(39.8)/279(48.4)	52(8.3)/245(39.1)/326(52.1)	0.13	
*FOXO3*	rs1935949	AA AG GG	59(10.2)/217(37.7)/299(51.9)	51(8.1)/234(37.4)/340(54.3)	0.41	
*FOXO3*	rs9486902	TT/CT/CC	1(0.1)/72(12.5)/501(87.0)	0/47(7.5)/578(92.3)	**0.008**	**0.096**
*AKT1*	rs3803304	CC/CG/GG	5(0.8)/82(14.2)/486(84.4)	7(1.1)/107(17.1)/500(79.9)	0.30	
*AKT1*	rs2494731	GG/CG/CC	63(10.9)/277(48.1)/236(41.0)	82(13.1)/268(42.8)/273(43.6)	0.18	
*AKT1*	rs2494732	TT/CT/CC	38(6.6)/223(38.7)/314(54.5)	55(8.8)/229(36.6)/339(54.2)	0.33	
*AKT1*	rs2498796	CC/CT/TT	73(12.7)/277(48.1)/225(39.1)	96(15.3)/278(44.4)/249(39.8)	0.30	
*AKT1*	rs2494738	GG/AG/AA	118(20.5)/289(50.2)/165(28.6)	145(23.2)/308(49.2)/169(27.0)	0.51	
*AKT1*	rs1130214	TT/GT/GG	9(1.5)/83(14.4)/478(83.0)	6(0.8)/99(15.8)509(81.3)	0.51	
*IGF-2R*	rs9456497	GG/AG/AA	133(23.1)/291(50.5)/152(26.4)	144(23.0)/322(51.4)/157(25.1)	0.89	

a Corrected *P, P* value after correction for multiple testing (*P*×12).

**Table 3:** Single nucleotide polymorphisms associated with human longevity

Gene	SNP (Minor allele)	Chr.	Position(bp)^a^	*P*-HWE^e^ (case/control)	MAF^d^ (case/control)	Allelic *P*^b^	Corrected *P*^c^	ORf (95% CI)^g^
*FOXO3*	rs13217795(T)	6	108974098	0.06/0.27	0.31/0.28	0.18	2.1	0.89(1.1-0.74)
*FOXO3*	rs 2464264(C)	6	108934461	0.16/0.52	0.31/0.28	0.19	2.2	0.89(1.1-0.74)
*FOXO3*	rs 9400239(C)	6	108977663	0.06/0.53	0.32/0.28	0.051	0.61	0.84(1.0-0.70)
*FOXO3*	rs 1935949(C)	6	108999287	0.04/0.23	0.29/0.27	0.21	2.5	0.89(1.1-0.75)
*FOXO3*	rs 9486902(C)	6	108878052	0.34/0.33	0.06/0.04	**0.002**	**0.024**	0.56(0.81-0.38)
*AKT1*	rs 3803304(C)	14	105239146	0.46/0.64	0.08/0.1	0.15	1.7	1.2(1.6-0.93)
*AKT1*	rs 2494731(C)	14	105237680	0.17/0.21	0.35/0.35	0.85	10	0.98(1.2-0.83)
*AKT1*	rs 2494732(T)	14	105239192	0.85/0.07	0.26/0.27	0.57	6.8	1.1(1.3-0.88)
*AKT1*	rs 2498796(C)	14	105243220	0.39/0.21	0.37/0.38	0.64	7.7	1.0(1.2-0.88)
*AKT1*	rs 2494738(G)	14	105246686	0.68/0.84	0.46/0.48	0.33	4	1.1(1.3-0.92)
*AKT1*	rs 1130214(T)	14	105259734	0.02/0.63	0.09/0.09	0.86	10	1.0(1.4-0.77)
*IGF-2R*	rs 9456497(A)	6	160443428	0.78/0.39	0.48/0.49	0.17	2.0	1.1(1.3-0.95)

a Position(bp), Genomic positions are according to NCBI build 37

b Allelic *P*, Allelic *P* has been adjust for gender

c Corrected *P*, *P* value after correction for multiple testing (*P* ×12).

d MAF, minor allele frequency

e *P*-HWE, *P*-value from Hardy-Weinberg Equilibrium test

^f^ OR, odds ratio;

g CI, confidence interval.

### Allele association analysis and gender stratification

Twelve SNPs from *FOXO3*, *AKT1* and *IGF-2R* were within Hardy–Weinberg equilibrium (HWE) in both case (*P* > 0.01, Table 3) and control groups (*P* > 0.01, Table 3). The minor allele frequencies (MAFs) of rs9486902 of *FOXO3* was greater in long-lived individuals than in the younger control population (*P* = 0.002, Table 3). After bonferroni correction, the significant associations remained existing (corrected *P* = 0.024, Table 3). When gender stratification is applied, in women, the MAFs of rs9486902 of *FOXO3* remained much higher in long-lived individuals than in the younger control population (*P* = 0.008, Table 5). Conversely, in men, rs9486902 did not show a statistic difference between the male subgroup (*P* = 0.073, Table 5).

Whether or not gender stratification was applied, (rs13217795, rs2764264, rs9400239, rs1935949) in *FOXO3*, all of *AKT1* and rs9456497 in *IGF-2R* did not show significant difference in MAFs (*P* = 0.22-0.057, Table 3) between case and control. Although the statistic differences didn't exist, the distribution trend of MAF of all SNPs from *FOXO3* was higher in case than in control in both genders.

**Table 4:** Genotype analysis of 12 SNP by four genetic models (Additive1 model, Additive1 model, Dominant model, Recessive model)

SNP ID (minor allele)	Additive 1		Additive 2		Dominant		Recessive	
	*P*^a^	OR^b^ (95% CI)^c^	*P*^a^	OR^b^ (95% CI)^c^	*P*^a^	OR^b^ (95% CI)^c^	*P*^a^	OR^b^ (95% CI)^c^
rs13217795	0.16	0.75(1.12-0.50)	0.61	0.94(1.20-0.74)	0.35	0.90(1.13-0.71)	0.18	0.77(0.65-1.25)
rs2764264	0.17	0.76(1.13-0.51)	0.57	0.93(1.19-0.73)	0.34	0.90(1.12-0.71)	0.20	0.78(0.77-1.21)
rs9400239	**0.04**	0.66(0.98-0.44)	0.46	0.91(1.16-0.72)	0.18	0.86(1.07-0.68)	**0.05**	0.68(0.82-1.27)
rs1935949	0.17	0.75(1.13-0.50)	0.67	0.95(1.21-0.75)	0.40	0.91(1.14-0.72)	0.19	0.77(0.83-1.58)
rs9486902	1.00		**0.003**	0.55(0.82-0.38)	**0.002**	0.55(0.81-0.37)	1.00	
rs3803304	0.68	1.28(4.07-0.40)	0.15	1.26(1.72-0.92)	0.14	1.26(1.71-0.93)	0.73	1.23(0.77-1.21)
rs2494731	0.56	1.12(1.62-0.77)	0.16	0.84(1.07-0.66)	0.33	0.89(1.12-0.71)	0.27	1.22(1.73-0.86)
rs2494732	0.23	1.32(2.05-0.845)	0.66	0.95(1.21-0.75)	1.00	1.00(1.26-0.80)	0.18	1.34(2.06-0.87)
rs2498796	0.35	1.18(1. 69-0.83)	0.47	0.91(1.17-0.71)	0.74	0.97(1.22-0.77)	0.20	0.96(1.72-0.89)
rs2494738	0.31	1.18(1.64-0.86)	0.81	1.03(1.35-0.79)	0.57	1.08(1.39-0.84)	0.29	1.02(1.53-0.88)
rs1130214	0.38	0.63(1.78-0.22)	0.47	1.13(1.55-0.82)	0.64	1.08(1.46-0.79)	0.36	1.15(1.75-0.22)
rs9456497	0.17	1.26(1.74-0.91)	0.24	1.18(1.57-0.90)	0.16	1.21(1.58-0.93)	0.40	0.93(1.46-0.86)

a *P*, *P* has been adjust for gender

b OR, odds ratio

c CI, confidence interval

Additive1 (AA versus BB); Additive2 (AB versus BB);

Dominant (AA+ AB versus BB); Recessive (AA versus AB+ BB)

A, minor allele; B, major allele.

**Table 5:** Gender effects on association of *FOXO3, AKT1* and *IGF-2R* with longevity

		MAF			
SNP	Gender	Cases/controls	OR	95%CI	*P*^a^
rs13217795	All	0.31/0.28	1.13	0.94-1.34	0.19
	Men	0.32/0.28	1.22	0.93-1.60	0.15
	Women	0.30/0.28	1.09	0.86-1.37	0.49
rs2764264	All	0.31/0.28	1.13	0.94-1.34	0.19
	Men	0.32/0.28	1.18	0.90-1.55	0.24
	Women	0.30/0.28	1.11	0.88-1.40	0.38
rs3803304	All	0.08/0.10	0.80	0.60-1.06	0.12
	Men	0.08/0.12	0.69	0.45-1.06	0.092
	Women	0.08/0.09	0.91	0.62-1.33	0.63
rs9400239	All	0.32/0.28	1.19	1.00-1.41	0.057
	Men	0.33/0.28	1.26	0.96-1.65	0.095
	Women	0.31/0.27	1.16	0.92-1.47	0.20
rs1935949	All	0.29/0.27	1.12	0.94-1.34	0.22
	Men	0.31/0.27	1.20	0.91-1.59	0.19
	Women	0.28/0.27	1.08	0.86-1.37	0.50
rs9486902	All	0.06/0.04	1.76	1.21-2.57	**0.003**
	Men	0.07/0.04	1.64	0.95-2.81	0.073
	Women	0.06/0.03	2.03	1.19-3.46	**0.008**
rs2494731	All	0.35/0.35	1.01	0.86-1.20	0.87
	Men	0.35/0.35	0.99	0.76-1.28	0.91
	Women	0.35/0.34	1.05	0.84-1.30	0.69
rs2494732	All	0.26/0.27	0.94	0.78-1.13	0.50
	Men	0.27/0.29	0.93	0.70-1.22	0.59
	Women	0.25/0.26	0.97	0.76-1.23	0.80
rs2498796	All	0.37/0.38	0.96	0.81-1.13	0.64
	Men	0.37/0.38	0.96	0.75-1.25	0.78
	Women	0.37/0.38	0.96	0.77-1.20	0.73
rs2494738	All	0.46/0.48	0.92	0.78-1.08	0.29
	Men	0.46/0.51	0.84	0.65-1.08	0.17
	Women	0.46/0.46	0.99	0.80-1.22	0.93
rs1130214	All	0.09/0.09	0.98	0.74-1.30	0.88
	Men	0.08/0.09	0.87	0.56-1.37	0.55
	Women	0.09/0.09	1.06	0.73-1.53	0.75
rs9456497	All	0.48/0.49	0.98	0.83-1.15	0.77
	Men	0.46/0.51	1.21	0.94-1.55	0.14
	Women	0.50/0.49	0.94	0.76-1.16	0.59

a *P*, *P* has not been adjust for gender

### Linkage disequilibrium and haplotype association analysis of *FOXO3* and *AKT1* gene

12 SNPs were analyzed LD and divided into two blocks (block1: rs13217795, rs2764264, rs9400239, rs1935949, and rs9486902, D'>0.86, 121kb; block2: rs3803304, rs2494731, rs2494732, rs2498796, and rs2494738 D'>0.71, 9kb.), according to their degree of association and genetic distance. We tested the five SNPs from *FOXO3* in this study using the program Haploview (Vision 4.2). We observed that haplotype TTCTT, CCTTC and CTCCT of *FOXO3* were shown to have a significant difference between case and control (P = 0.006, OR= 0.57 (0.38-0.86); 2.78×10^−5^ OR= 8.7 (2.6-29); 4.68×10^−6^ OR=23 (3.2-1.7 E-2)

Table 6). Among them, while the haplotype TTCTT was the protective haplotype, the haplotype CCTTC and CTCCT were the risk haplotypes (Table 6). Furthermore, six SNPs of *AKT1* were also tested in the present study. We found that haplotype GGTCGG and GGTCAG have a significant difference between case and control (*P* = 0.003, OR= 1.4(1.1-1.7); 0.005, OR= 0.56(0.37-0.85) Table 7). Among them, while the haplotype GGTCAG was the protect haplotype, the haplotype GGTCGG was the risk haplotype (Table 7).

**Table 6:** *FOXO3* haplotype associated with human longevity in a Han Chinese population

Haplotype*	Frequency	Case, control frequencies	Chi-square	*P*-value	OR(95% CI)
CCTCC	0.671	0.631,0.669	0.058	0.81	
CTCTT	0.205	0.200,0.210	0.318	0.57	
TTCTT	0.042	0.031,0.054	7.71	0.006	0.57(0.38-0.86)
CTCTC	0.021	0.016,0.025	2.191	0.14	
CCTTC	0.013	0.022,0.003	17.561	2.8E-5	8.7(2.6-29)
CTCCT	0.010	0.020,0.001	20.965	4.7E-6	23 (3.2-1.7 E-2)

* The haplotypes were generated from SNPs rs13217795, rs2764264, rs9400239, rs1935949, rs9486902, respectively.

**Table 7:** *AKT1* haplotype associated with human longevity in a Han Chinese population

Haplotype*	Frequency	Case, control frequencies	Chi-square	*P*-value	OR(95% CI)
CGCTAG	0.435	0.432,0.437	0.066	0.80	
GGTCGG	0.201	0.224,0.176	8.644	0.003	1.4(1.1-1.7)
CCCTGG	0.078	0.085,0.070	1.981	0.16	
CGCTGG	0.056	0.049,0.063	2.073	0.15	
GGCCGG	0.043	0.038,0.047	1.077	0.30	
GGTCAG	0.039	0.029,0.051	7.754	0.005	0.56(0.37-0.85)
GGCCGT	0.034	0.032,0.037	0.568	0.45	
CGCTAT	0.031	0.034,0.028	0.629	0.43	
CGCCGG	0.022	0.020,0.023	0.235	0.63	
GGTCGT	0.012	0.013,0.011	0.188	0.67	

* The haplotypes were generated from SNPs rs3803304, rs2494731, rs2494732, rs2498796, rs2494738, rs1130214.

**Table 8:** Comparison for prevalence of diabetes between longevity and normal people reported

	People with prediabetes	Normal people	Total	Prevalence rate (%)	*P*
Case	42	534	576	7.29	0.001
Control*	11444	87214	98658	11.6	

* Data from Xu et al^[18]^.

**Table 9:** Comparison for prevalence of prediabetes between longevity and normal people reported

	People with prediabetes	Normal people	Total	Prevalence rate (%)	*P*
Case	137	439	576	23.78	2.3×10-26
Control*	49428	49230	98658	50.1	

* Data from Xu et al^[18]^.

## DISCUSSION

In this study, we attempt to determine whether *FOXO3*, *AKT1* and *IGF-2R* are associated with human longevity in a Han Chinese population. We used a case-control study to examine the relationship between the 12 SNPs of *FOXO3*, *AKT1* and *IGF-2R* with human longevity in a Han Chinese population. Then we compared the prevalence of diabetes of long-lived individuals in our study with common adult population reported by Xu et al. [22].

When comparing genotype frequencies, we found that rs9486902 in *FOXO3* was significantly different between the longevity group and the younger control in the present study. However, after bonferroni correction, the significant associations no longer existed, suggesting that this locus was unlikely to be associated with longevity in the present study. In the following we used different genetic models (Table 4). Rs9486902 showed highly significant association in Addictive 2 and Dominant models. Rs9400239 showed borderline significant association in Addictive 1 and Recessive models Table 4). But after the multiple testing, the significant associations no longer existed, suggesting that these loci were not associated with longevity in the present study.

When performing association studies by comparing allele frequencies between long-lived individuals and younger individuals, we found that rs9486902 in *FOXO3* was significantly associated with longevity in both genders combined in this study. For rs9486902, the risk allele frequency was 0.06 in the cases and 0.04 in the controls (Table 3). The T allele in rs9486902 of *FOXO3* was higher in the long-lived individuals than that in the younger controls. The study results were consistent with Tan et al. [24] and Soerensen et al's report in Danish population [9]. Lineages of long-lived individuals were classified according to Y chromosome haplogroups lineage distribution. N* is mainly distributed on both side of the Ural mountains, the north of the Scandinavia, the north of eastern Europe,(Russia, Finland, Hungary, Estonia, Sami etc), the southwest of China. Of note, the genetic variation of similar lineage distribution has approximate consistency. Thus, we propose a hypothesis that the principle of Y chromosome haplogroups is approximately consistent with autosomal genetic variation.

Further analysis using haploview4.2 software yielded the risk haplotype CCTTC and CTCCT of *FOXO3* from five SNPs proved to be significantly different between long-lived individuals and younger controls. An individual with these risk haplotypes has 8.7-fold and 23-fold increased likelihood of longevity (Table 6). The risk haplotype GGTCGG of *AKT1* from six SNPs conferred a 1.35-fold increased likelihood of longevity (Table 7).

Previously, willcox et al. [6] reported that the single nucleotide polymorphisms of the *FOXO3* gene (rs2802292, rs2764264, rs1327795) were associated with ethnic Japanese, suggesting that these SNPs were potentially susceptible polymorphisms of human longevity. However, these variations were not replicated in the present study. The possible reasons as follows: first, it might be due to the genetic heterogeneity of human longevity among different populations. Willcox et al's report referred to males only. Thus, we can classify population by Y chromosome haplogroups lineage distribution. According to the haplogrouping and nomenclature system of The Y Chromosome Consortium (YCC), Japanese males mainly belong to haplogroups C, D2, D2a1, O2b*, O2b1, and O3*[23, 25]. O3* haplogroups account for only 20% in Japanese males, whereas, more than 80% of the Chinese males are O3* haplogroups. Second, these SNPs might be small genetic effect sizes to human longevity in a Han Chinese population. Third, it might be the mean age of 97.9 in willcox et al's report, however, we have only 11 long-lived individuals >100 years, mean age 92.8 in the present study. Flachsbart et al. [13] and Deelen j et al. [26]also showed that the effects of *FOXO3* on longevity were most prominent in centenarians. Fourth, it is possible that a small sample size generates insufficient statistical power.

A study by Terry et al. [1] reported that long-lived individuals (nonagenarians and centenarians) will delay or escape from age-related disease, such as hypertension, coronary heart disease [27], cancer, and type 2 diabetes [28]. Among them, diabetes is a high risk factor for morbidity and mortality all over the world [29]. In this study, we further tested the hemoglobin A1c level in longevity group. According to the 2010 American Diabetes Association criteria [30], the estimated prevalence of diabetes in this study's longevity group was 7.29%(42 cases) and the prevalence of prediabetes was 23.78% (137 cases). Considering the fact that younger control individuals who we selected were mean age: 33 years, range: 20–40 years old. These subjects were not the vulnerable group of diabetes. Thus, comparing the prevalence of diabetes in long-lived individuals with younger control individuals has no clinical significance. Referring to previous reports, Xu et al. [22]reported that the estimated prevalence of diabetes was 11.6% in a representative population of Chinese adults and the prevalence of prediabetes was 50.1%. Because the same sampling methods, detecting instrument, and diagnostic criteria were used in the 2 investigations, suggesting that the 2 investigations may be comparable. we compared our study of long-lived individuals with the result described by Xu et al. [22]. The estimated prevalence of diabetes and prediabetes of long-lived individuals was significantly lower (P = 0.001, 2.3×10^−26^ using chi-square test) than common adult populations. In addition, we found that rs9486902 in *FOXO3* gene is associated with human longevity in both genders in this study. The future challenge is to test whether rs9486902 in *FOXO3* is involved in diabetes and increases susceptibility to diabetes and prediabetes. Therefore, the search for longevity-associated genes should lead not only to an understanding of the fundamental mechanisms of human longevity, but also to the identification of new potential targets beneficial for the treatment of diabetes. It is believed that diabetes is a complex disorder caused by the interaction of multiple genetic and environmental risk factors. Further replication studies are needed to clarify the effect of living conditions and selection pressure changes on longevity.

Recently, Pawlikowska et al. [12] showed that the polymorphisms of the *AKT1* gene (rs 3803304) was significantly different between human longevity and controls in Ashkenazi Jewish Centenarians (adjusted *P* = 0.043). However, Nygaard et al. [16] showed that *AKT1* is unlikely associated with human longevity in German and Danish cohorts. Consistent with results in German and Danish populations, we did not find that rs3803304 in *AKT1* gene was associated with human longevity in a Han Chinese population. However, we did not confirm *AKT1* as a human longevity gene in populations of a Han Chinese, because our selection of SNPs in *AKT1* is not complete. Nojima et al. [31] reported that Haploinsufficiency of *AKT1* prolongs the lifespan of mice, therefore, further studies are need to confirm that *AKT1* is associated with human longevity. Rs3803304 in *AKT1* located in an intron, 70 bp away from a exon-intron boundary and in an area of high-predicted regulatory potential [12, 32], a CpG island is also found here, RNA signal at this intron\exon border encompasses rs3803304. It is possible that rs3803304 disrupts an RNA regulatory mechanism. It might be a candidate regulatory for influencing RNA splicing by microRNA, siRNA or lncRNA. The future challenge is to investigate whether this SNP rs3803304 also pose effects on gene expression. First, cloning this locus and transfect fibroblast [33, 34]. Second, to investigate whether this SNP also pose effects on *DNMT1* (DNA methyltransferase 1) [35], leading to epigenetic changes. It was previously shown that *IGF-2R* gene was significantly associated with human hepatic carcinoma [36]. A study composed of 1089 long-lived individuals and 736 younger controls demonstrated that rs9456497 in *IGF-2R* was significantly associated with human longevity in Danish subjects [18]. However, this variation was not replicated in our study, suggesting that this locus/gene was not significantly associated with human longevity in Han Chinese population in the region of Sichuan.

In summary, we genotyped 12 SNPs from *FOXO3*, *AKT1* and *IGF-2R* gene in a Han Chinese population composed of 576 long-lived individuals and 626 younger controls. Our results showed one SNP (rs9486902 in *FOXO3*) was significantly associated with human longevity in both genders combined in this study. The six SNPs in *AKT1* gene and rs9456497 in *IGF-2R* gene are not significantly associated with human longevity in this study. This is the first investigation to analyze a possible association between *AKT1* and *IGF-2R*, respectively, with human longevity in Han Chinese population in Sichuan region. Our results suggest that *AKT1* and *IGF-2R* are not universally human longevity genes. The estimated prevalence of diabetes and prediabetes of long-lived individuals were significantly lower than common adult population in Han Chinese population in Sichuan region.

## MATERIALS AND METHODS

### Study subjects

The studied population included 576 long-lived individuals (nonagenarians and centenarians) (range 90-108 years, mean age 92.3 years, women proportion of 61.6% in all case). The younger control population were 626 unrelated individuals (range 20-40 years, mean age 32 years, women proportion of 55.0% in all control) (Table 1). The procedures were approved by the Institutional Review Boards of the Sichuan Academy of Medical Sciences & Sichuan Provincial People's Hospital. All individuals gave written informed consent to the study. Diagnosis of diabetes and prediabetes was based on the 2010 American Diabetes Association criteria. The basic information about long-lived individuals and younger control individuals is listed in Table 1.

### SNPs selection and genotyping

Based on comprehensive literature/data base searches in different candidate longevity genes, we have selected longevity related gene/loci in this study. According to the HapMap Phase II+III (Feb.2009) of CHB database(Han Chinese in Beijing, China), the SNPs rs13217795, rs2764264, rs9400239, rs1935949, and rs9486902 were selected for *FOXO3*, the SNPs rs3803304, rs2494731, rs2494732, rs2498796, rs2494738, rs1130214 for *AKT1*, the SNP rs9456497 for *IGF-2R*.” [6, 9, 16, 18, 24] (Table 2). DNA was extracted from peripheral blood leukocytes, using serial phenol/chloroform extraction and ethanol precipitation. Genotyping of the Han Chinese population was performed using the dye terminator-based SNaPshot method (Applied Biosystems, Foster City, CA). The SNP analysis was performed on the ABI 3130XL genetic analyzer (Applied Biosystems). All of the SNPs reported in this study had a genotyping success rate 98 percent and accuracy assessed by random genotyping of 15 percent of the samples in the subject group.

### Hemoglobin A1c measurement

Whole blood was collected in EDTA-treated tubes, and the concentration of hemoglobin A1c level was determined by high-performance liquid chromatography (HPLC) using the VARIANT II Hemoglobin Testing System (Bio-Rad Laboratories) at the central laboratory in Sichuan Academy of Medical Sciences and Sichuan Provincial People's Hospital. According to the 2010 American Diabetes Association criteria, diabetes was defined as HbA1c concentration of 6.5% or more. Prediabetes or categories of increased risk of diabetes were defined as HbA1c concentrations between 5.7% and 6.4% in participants without a prior diabetes diagnosis.

### Statistical methods

The Hardy-Weinberg equilibrium (HWE) for 12 SNPs between the case and control was assessed by the χ2 test. The P values of the SNPs were calculated by an additive model. Differences in allele and genotype distribution between cases (long-lived individuals) and controls (younger population) were tested using logistic regression with adjustment for gender. The haplotype blocks, the D'values and r^2^values for all pairs of SNPs were calculated by using software Haploview Vision 4.2. All statistical analyses were carried out using the software SPSS version 17.0 (SPSS Inc., Chicago, IL). In order to further investigate the association between *FOXO3*, *AKT1* and *IGF-2R* with longevity, we applied four statistical models including additive 1 model, additive 2 model, dominant model and recessive model. Four statistical models were defined as 1(AA+ AB) versus 0(BB) for dominant, 1(AA) versus 0(AB+ BB) for recessive, 0(AA) versus 1(BB) for additive1, and 0(AB) versus 1(BB) for additive2 (A: minor allele; B: major allele).
